# Further interceptions of the Neotropical fungus gnat *Sciophilafractinervis* Edwards, 1940 (Diptera, Mycetophilidae) in Britain with comments and observations on its biology and spread

**DOI:** 10.3897/BDJ.10.e94812

**Published:** 2022-12-14

**Authors:** Rob J. Deady, Mark A. Delaney, Eleanor Jones, Peter J. Chandler

**Affiliations:** 1 Fera Science Ltd, York Biotech Campus, York, United Kingdom Fera Science Ltd, York Biotech Campus York United Kingdom; 2 606B Berryfield Lane, Melksham, Wilts SN12 6EL, United Kingdom 606B Berryfield Lane, Melksham Wilts SN12 6EL United Kingdom

**Keywords:** *Sciophila*, Mycetophilidae, Fungus gnat, glasshouse, Neotropical

## Abstract

From 2020 onwards, several specimens of the Neotropical fungus gnat *Sciophilafractinervis* (Edwards, 1940) have been intercepted by Fera Science Ltd. on or near plant material in the United Kingdom originating from nurseries and glasshouses at four separate locations: Preston, Lancashire; Chichester, West Sussex; East Riding of Yorkshire; and Middlesbrough, North Yorkshire, as well as a single interception from the Netherlands. Gnat interceptions were associated with a wide range of plant species: *Ficusbenjamina*, *Ficuselastica*, Dracaenareflexavar.angustifolia, *Origanumvulgare*, *Rosmarinusofficinalis*, *Thymusvulgaris*, *Impatienshawkeri* (*Impatiens* New Guinea hybrids), *Chrysanthemum*, as well as *Fragariavesca* (var. *Lusa*). The species does not appear to be doing any damage to affected plants with growing conditions likely promoting multiplication under protection. The larvae likely feed on spores (generally saprophytic) adhering to webs they erect on the soil and around the base of plants with spores likely originating from the growing medium and plants. Their spread is likely facilitated by movement of the growing medium or plant material where pupae are suspended in the lower stem or leaf axils.

An account of the various interceptions is provided, as well as images of the different life-stages with a brief discussion of *Sciophilacincticornis* Edwards, 1940, its relationship with *Sciophilafractinervis* and further evidence of instability in vein R_2+3_ in the *Sciophila* genus.

## Introduction

**Figure 1.**
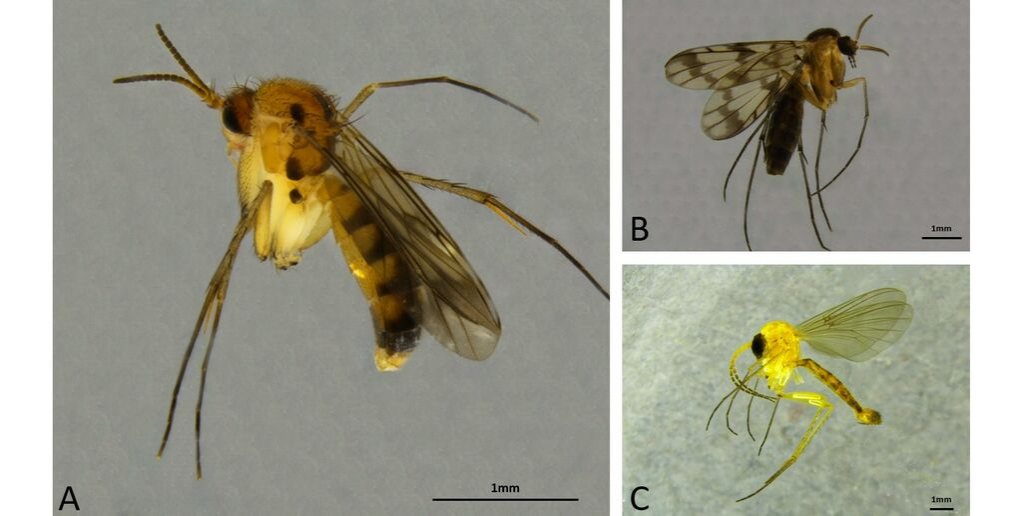
Very few gnats are intercepted on produce into and out of the United Kingdom with few also detected on plants grown in nurseries. Above are examples of some of the species that have been confirmed. **A** *Leia* sp. intercepted on *Zingiber* from China. **B** *Proceroplatustrinidadensis* Lane, 1960 intercepted on *Monsteradeliciosa* that originated from the Netherlands. **C** *Sciophilacorlutea* intercepted on *Prunuspersica* from Spain (likely adventitious).

Fera Science Ltd. provides diagnostic support for the plant health service in England and Wales. The Plant Health and Seeds Inspectorate (PHSI) submit samples that they supect may be regulated or non-native to Fera for identification. Apart from gall midges (Cecidomyiidae) and darkwing fungus gnats (Sciaridae) (common pests in greenhouses), other members of the Sciaroidea, like the true fungus gnats (Mycetophilidae) and predatory fungus gnats (Keroplatidae) (such as *Proceroplatustrinidadensis*, Lane 1960, Fig. 1B), are rare interceptions. The two main reasons for this are: 1) There are no regulated species of fungus gnats and 2) fungus gnats are generally sporophagous or mycetophagous as the name suggests and, thus, PHSI inspectors are less likely to submit them. This assumption, while generally true, leads to specimens only being identified as "Mycetophilidae" or "Keroplatidae" or specimens are frequently left at generic level. In the last 10 years, *Sciophilacorlutea* Chandler & Blasco-Zumeta, 2001 (adventive on *Prunuspersica* ex. Spain, Fig. 1C) has been intercepted, as well as an unidentifiable female of a *Leia* species on *Zingiber* ex. China (Fig. 1A).

**Figure 2.**
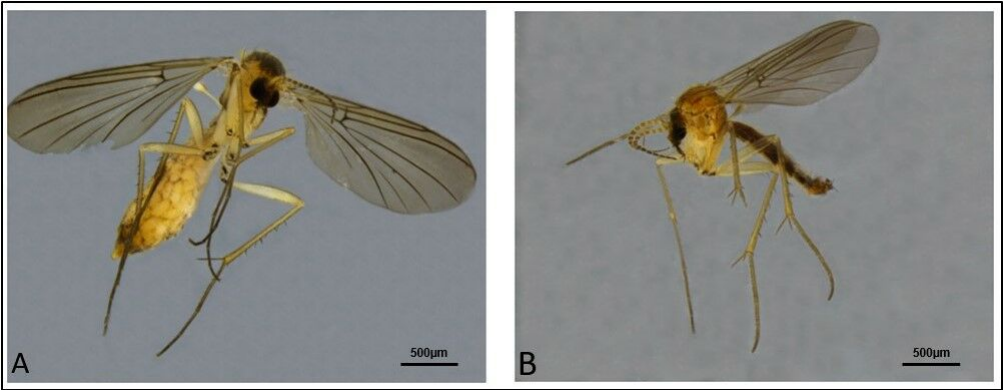
Adult specimens of *Sciophilafractinervis*. **A** Gravid female *Sciophilafractinervis* (atypical form lacking R_2+3_ vein, further reinforcing the instability of this character); **B** A typical male *Sciophilafractinervis* (R_2+3_ vein present creating a radial cell).

Aside from these, one species that has been encountered more than others in recent times is the Neotropical fungus gnat *Sciophilafractinervis* (Edwards, 1940) (Fig. 2A, B), described from the south of Brazil and not currently recorded elsewhere in South America (Chandler 2022).

The species was first recorded in Britain over 10 years ago by Peter Chandler from specimens recovered from a glasshouse in Warwickshire and apparently associated with Lisianthus (*Eustoma grandiflorum)* and potentially Christmas cacti (*Epiphyllum*) (Chandler 2010). Interestingly, around the same time as Chandler’s confirmation, Fera Science Ltd. received two adults and two larvae of suspected *S.fractinervis* from Warwickshire, except only a single adult female was reared with an overall habitus resembling that of *S.fractinervis*. At the time, it was noted that the individual resembled “the British species *S.interrupta* in the form of the posterior fork on the wing” (basal abbreviation of the posterior fork, CuA1 Fig. 2A, B), a character also shared with *S.cincticornis*), but was “too light in colour to be that species”. Without a male at the time, confirmation was impossible. Since then, the status of *S.fractinervis* in the U.K. has remained largely a mystery and its biology enigmatic. A recent increase in interceptions led to the writing of this article.

**Figure 3.**
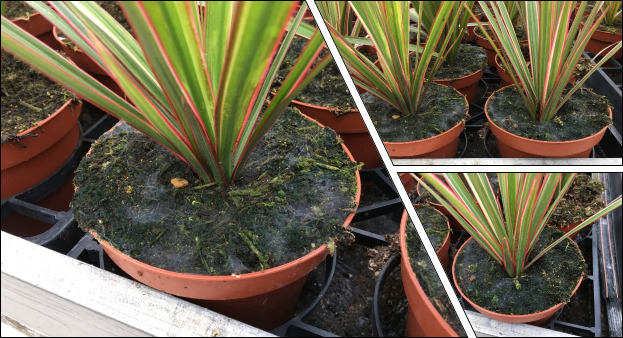
Showing the fine, filamentous webbing produced from labial glands of *Sciophilafractinervis* larvae in and around *Dracaena* plants from Sri Lanka via the Netherlands.

**Figure 4.**
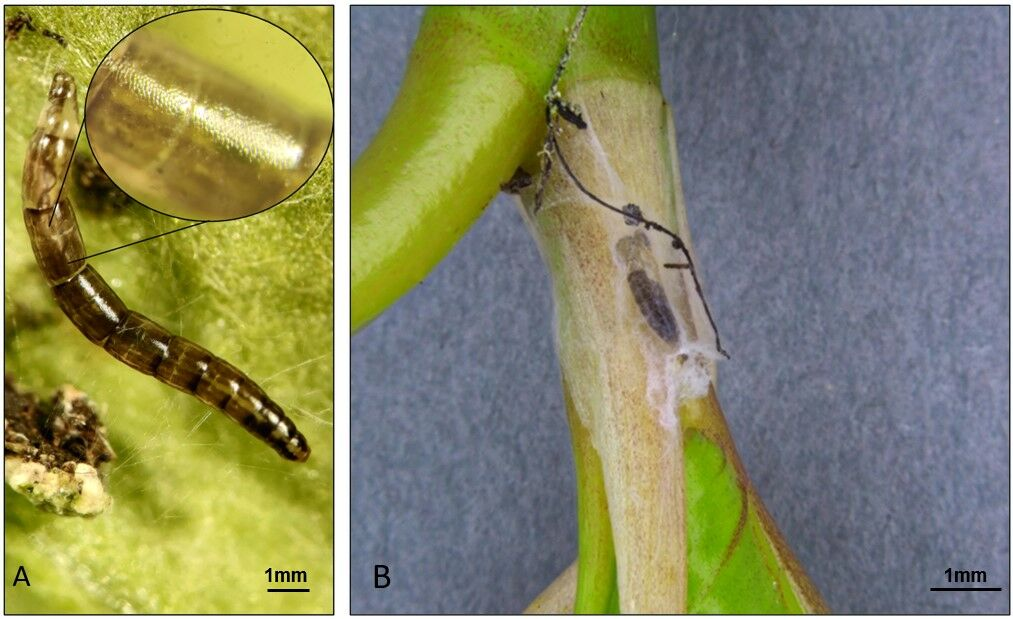
The immature lifestages of *Sciophilafractinervis*. **A** The larva of *Sciophilafractinervis* with close-up of cuticular ultrastructure made up of a mesh-like network on a decaying *Chrysanthemum* leaf. **B** The suspended pupa of *Sciophilafractinervis* surrounded by fine, filamentous webbing on Dracaena.

**Figure 5.**
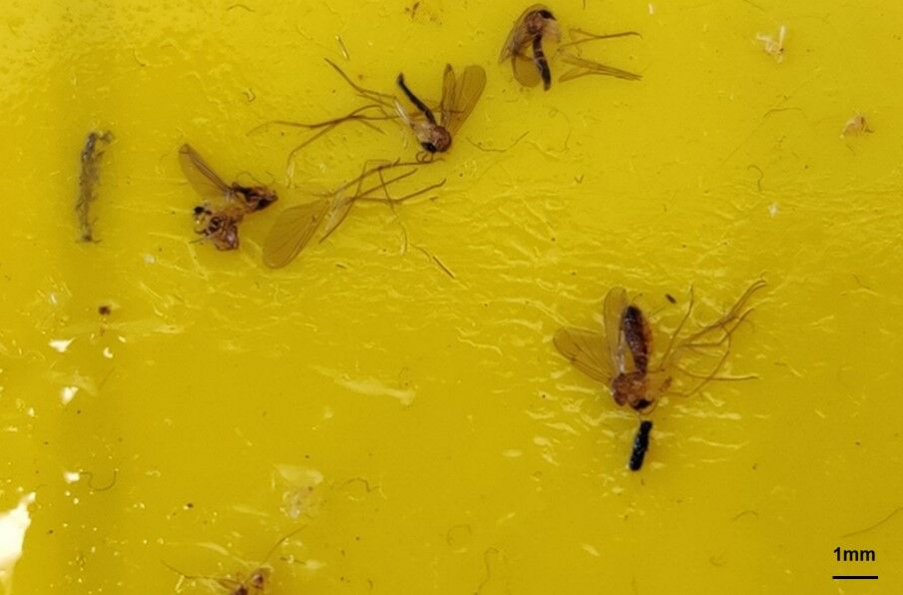
Numerous *Sciophilafractinervis* sampled using sticky traps during standard monitoring for *Bemisiatabaci* in a *Fragaria* crop- Preston, Lancashire, U.K.

1. In November 2020, the first gnat specimens started arriving in the labs. Reports of insect infestation was reported in glasshouses in Chichester, West Sussex in apparent association with a proportion of (ca. 500) *Ficusbenjamina*, *Ficuselastica* and Dracaenareflexavar.angustifolia (Syn. *D. marginata)* plants with some plants originating from Sri Lanka and others from the Netherlands. Initial suspicions by growers and inspectors were of “Orchid worm” (various Keroplatidae known to be an issue in greenhouses in the Netherlands, such as *Lyprauta* spp., *Proceroplatustrinidadensis*) or an unidentified tortrix moth due to webbing networks erected at the base and further up the plants, especially near the leaf axils. The PHSI (Plant Health and Seed Inspectorate) submitted symptomatic plants. *Dracaena* plants arrived to the labs with a thin, filamentous layer of webbing strewn across the potting medium’s surface at the base of the plants, as well as the lower stem and leaf axils (Fig. 3). To the casual eye, the webbing resembled saprophytic fungi or actinomyces bacteria that sometimes colonise the surface of potting media in greenhouses. A Nematoceran larva (Fig. 4A) was seen moving about on webbing at the base of the plant and a further pupa was suspended in webbing higher up in the plant (Fig. 4B).
2. Two female gnat specimens arrived in January 2021 from glasshouses in East Riding of Yorkshire involving ca. 928 plants with an estimate of 25% of plants affected. The crop consisted of mixed herbs (*Rosmarinusofficinalis*, *Thymusvulgaris* and *Origanumvulgare)* originating from Italy. The specimens were mixed in amongst frequently detected glasshouse insects, such as Ligurian leafhoppers (*Eupteryxdecemnotata* Rey, 1891), Onion thrips (*Thripstabaci* Lindeman, 1889), dark-wing fungus gnats (*Bradysia* spp.), as well as various Collembola and predatory Acarina.
3. A third interesting interception on 8 May 2021 involved numerous gnats, both males and females, being found on sticky traps in a nursery in Preston, Lancashire, U.K. (Fig. 5). The sticky traps were being used to monitor pests in and around a *Fragaria* crop.
4. A fourth finding on 25 May 2022 was of a Nematoceran pupa adhering to a leaf of an *Impatiens* New Guinea hybrid from a nursery in Middlesbrough, North Yorkshire, U.K.
5. The final finding was on 19 October, 2022 where several larvae and a live adult were found in association with *Chrysanthemum* from the Netherlands where larvae resided under lower leaves and axils of the plant that were draped over the soil.

## Materials and methods

All gnat specimens were initially examined using a stereomicroscope (Leica 205C) with a Schott KL1500 LCD light source. All live larvae were examined by gently rolling them between the lid of a 150 mm × 15 mm Petri dish and the lid of a 60 mm × 15 mm Petri dish so as to view all characters required to identify the larva. Care was taken to avoid excessive pressure being applied to the specimens. Larvae were provisionally identified using a combination of Madwar (1937), Hutson et al. (1980) and Zaitzev (1982a)

Symptomatic plants (with the larvae, pupae etc.) received were enclosed in sealed containers with breathing holes (sealed with mesh) and then placed in an incubator (240L Sanyo Co2 Incubator MIR-253) subsequent to visual examination where possible so as to rear these specimens to adulthood. In the case of the Middlesbrough finding, the pupa was placed on filter paper, in a Petri dish and sealed.

For adult gnat specimens submitted on sticky traps as in the case of the Preston finding, extraction and cleaning of the glue followed Appendix 1 (Protocol for removal of adult whitefly from sticky traps) in Malumphy et al. (2010). All adult gnats, whether successfully reared or extracted from sticky traps, were then slide-mounted unless they were destined for DNA sequencing. Males were ultimately needed for species determination. Their abdomens were removed and genitalia dissected away from the abdomen with dissecting needles and placed in an embryo dish with a solution of 10% potassium hydroxide (KOH), covered with a glass lid and placed on a hotplate at 80°C for 20 minutes to allow for maceration of the soft body contents. The genitalia were then gently pressed/palpated with a micro-spatula to expel any residual soft tissue. After neutralising with glacial acetic acid (CH₃COOH), the genitalia were immersed in 70% ethanol and further tissue and cuticle were removed exposing the genitalia. Genitalia were then transferred to absolute ethanol for 5 minutes, then clove oil before being mounted on a slide in Canada Balsam with an 11 mm coverslip along with the rest of the specimen (head, wings, legs and thorax). Adults were identified to species (if male) and genus (if female) using a combination of Edwards (1940), Hutson et al. (1980), Chandler (2006), Chandler and Pijnakker (2009) and Søli (2017). Specimens identified morphologically were deposited in the FERA plant health entomological reference collection.

Several strands of silk laid down by gnat larvae in the Dutch *Chrysanthemum* sample were examined for the presence of fungi. Strands were plated up on sterilised Petri dishes of PDA (Potato Dextrose Agar), incubated and identified on the basis of cultural, microscopic and morphological characteristics if possible.

**Molecular methods**

Two adult female gnats originating from East Riding of Yorkshire were sequenced for the COI DNA barcode (Hebert et al. 2003), as females cannot be identified morphologically and also due to a clear difference in wing morphology between the specimens. The hind legs of each specimen were removed, placed in 1.5 ml Eppendorf containers and stored in a freezer at -18°C. DNA was extracted from each leg separately (4 samples in total, from 2 individuals) using the QIAGEN Blood and Tissue Kit, following the manufacturers' recommended protocol. Samples were amplified by PCR using the primers C1-JF-1718 and C1-NR-2191 (Simon et al. 1994) and MiFi Mix (Bioline, UK) PCR reagent master mix. PCR amplicons were cleaned up using ExoSAP-IT Express (ThermoFisher) and sequenced using the PCR primers by Sanger sequencing at Eurofins Genomics, Ebersberg, Germany.

Any fungi not identified using morphological means were identified via DNA sequencing in the Btub and ITS gene regions.

## Results

Larvae examined from Chichester, West Sussex exhibited all the characteristics of a *Sciophila* spp. larva. Under-developed antennae, well-developed maxillary palps, peripneustic in terms of spiracular layout and locomotory hooks being visible on the ventral surface of the body (presumably used to adhere to the webbing upon which it moved). It took 10 days for a single male fungus gnat to be reared from specimens on plants stored in the incubator and this was determined as *Sciophilafractinervis* (Edwards, 1940), a Neotropical species that is established in nurseries in the Netherlands (Chandler and Pijnakker 2009).

Specimens from East Riding of Yorkshire (both female) were confirmed as *Sciophila* spp. and one close to *S.fractinervis* with the other close to *S.cincticornis* (Edwards, 1940). The suspected *S.cincticornis* generally followed the description by Edwards (1940) and Chandler and Pijnakker (2009) with vein R_4_ (now to be interpreted as R_2+3_ following Søli (2017)) absent, flagellomeres slightly yellow on basal two fifths to half and abbreviated anterior branch of the posterior fork (CuA1) (Fig. 2A, B). The other specimen was more typical of *Sciophilafractinervis* with the R_2+3_ vein present, creating a radial cell. All other instances of suspect *S.fractinervis* were confirmed morphologically. Several fungi were present on the isolation plates cultured from strands created by larvae intercepted amongst *Chrysanthemum* from the Netherlands. The majority were saprophytic with an unknown *Acremonium*- like fungus being the most dominant. *Alternaria*, *Penicillium* and a *Mucor* species were also present.

**Molecular results**

COI DNA sequences were generated for the two reference samples, with two independent sequences generated per sample. Final sequence lengths were between 444 and 457 base pairs long. Both specimens had the same haplotype (i.e. they share the same DNA sequence), indicating they were likely the same species. There were no COI reference sequences for *Sciophilacincticornis* or *Sciophilafractinervis* on either the BOLD (Ratnasingham and Hebert 2007) or GenBank (Benson et al. 2012) public databases. A BLAST search against the GenBank nucleotide database found close matches (up to 99% pairwise similarity, closest match MG104750.1) to *Sciophila* sequences that were not identified to species. Similarly, a search against the BOLD database found close matches (up to 100% pairwise similarity) to *Sciophila* sequences not identified to species. These were in BOLD BIN BOLD:ABV9018 (Ratnasingham and Hebert 2013) and it seems likey that this BIN either corresponds to the species *S.fractinervis* or contains it. Sequences will be uploaded to GenBank and BOLD [accessions to follow, contact author for details]. The unknown "*Acremonium*-like fungus" was sequenced and identified in the Btub region but only to genus level: a *Plectosphaerella* species. In ITS, one of the cultures matched 100% to two *Plectosphaerella* species (*P.pauciseptata* and *P.cucumerina*); the other culture also matched 100% to two species – *P.plurivora* and *P.niemeijerarum*. Unfortunately the sequencing results from these gene regions did not allow us to distinguish them further.

## Discussion

Not a great deal is known about the biology of the *Sciophila* genus nor the larval diet (Kurina 2020). The larvae of most species tend to live on, within or on the underside of the sporophore/fruiting bodies of mainly wood-associated or lignicolous fungus species where they construct webs and feed on spores (Zaitzev 1979, Zaitzev 1982a, Zaitzev 1982b Zaitzev 1982b, Falk and Chandler 2005, Chandler 2006, Ševčík 2010, Bouchard and Bouchard-Madrelle 2010, Jakovlev 2011). They may also be found in association with fungal mycelia (Zaitzev 1979, Zaitzev 1982a, Zaitzev 1982b, Chandler and Pijnakker 2009) particularly that which is found in association with deadwood (Zaitzev 1979, Zaitzev 1982a, Bechev and Koç 2006). The larvae of *S.fractinervis*, as in other members of the Sciophilinae, tend to be enclosed in a mucous tube or “delicate tube of mucilage” created from labial glands around the mouth (Madwar 1937, Zaitzev 1982b) which we observed often giving larvae a shiny appearance (Fig. 4A). Coupled with this, the cuticular ultrastructure of larvae appeared to have a fine mesh/reticulate network. This network likely acts as a plastron of sorts that, amongst other things, aids respiration.

**Figure 6.**
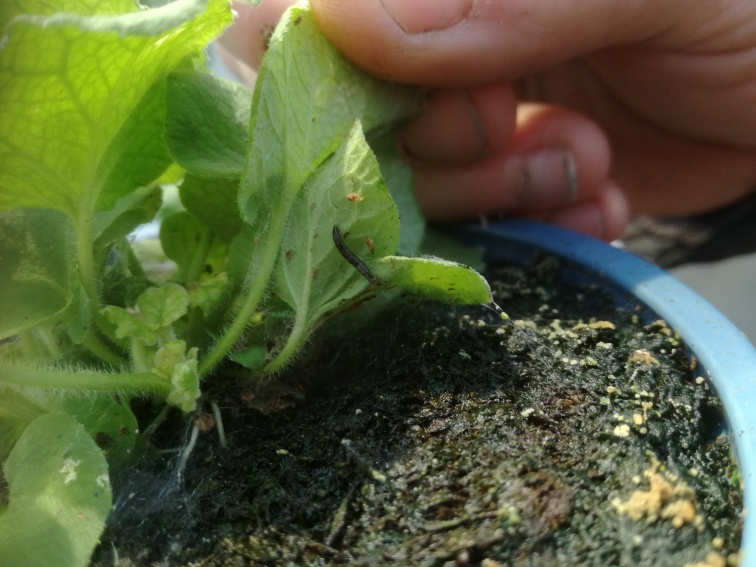
Neil Helyer (Fargro Ltd.) lifting the leaf of a *Primulaobconica* plant in a nursery in Ireland to reveal a "*Sciophila*-like" larva on the underside of the plant. Note the webbing again near the base of the plant.

Growers, in many of the instances where *S.fractinervis* was found, noticed webbing forming on the compost surface (Figs 3, 6) even prior to planting. It is apparent that webbing produced from labial glands situated in the heads of larvae of *S.fractinervis* is of great importance to their life-history. The scaffolding-like webs appear to help the larvae pupate in a dry place, while also acting as a potential barrier protecting against predation (Fig. 4B). Any sudden vibrations on the webs or "predator-like" movements towards larvae of *S.fractinervis* in the lab, elicited a rapid retreat response. Potting media used in horticulture are frequently enriched with microbial biostimulants that can promote mycorrhizal fungus. It is likely that the larvae of *S.fractinervis* potentially feed on spores stuck to their webs that originate from the potting medium itself below the webs, but also fungi residing on the plants themselves. It is evident that the surface of webs become covered in airborne spores in glasshouses, generally saprophytic species. Some *Plectosphaerella* species are known plant pathogens (*P.cucumerina*). Many are associated with soil and plant debris. There are also species isolated from living plants. Further gut analysis of larvae is needed to ascertain whether larvae are opportunists or select certain species.

Other members of the Sciophilinae have been known to use webbing networks to effectively snare and feed on smaller invertebrates, but such feeding behaviour was not observed here or in the account by Chandler (2010). As far as the adults are concerned in terms of diagnostics, it would appear that the presence of R_2+3_ in *Sciophilafractinervis* may be an unstable character like other *Sciophila* spp. in the world as Chandler and Pijnakker (2009) suggest.

## Conclusions

To conclude, it appears that *S.fractinervis* is here to stay in Britain with interceptions and submissions to the lab on the rise as those in industry recognise symptoms of presence, larvae and the adults. More work ought to be carried out to ascertain the larval diet in an ex-situ context; however, saprophytic fungi likely sustain the larvae in a horticultural setting.

It is uncertain as to where the above-documented occurrences of the species originated. It is most likely the Netherlands, but in some instances, plants also originated from Costa Rica and Denmark. It is likely that this enigmatic species is more widespread than first realised especially in Europe. There is evidence that it is in the Republic of Ireland. Photos (Fig. 6) were submitted at the end of 2020 to the corresponding author of a "*Sciophila*-like" larva and accompanying webbing on *Primulaobconica* from a nursery between Cork and Dublin with anecdotal evidence of similar larvae being found on *Poinsettia* the previous year. Unfortunately from photos, species determination was not possible.

*S.fractinervis* does not appear to be doing any damage to the great many plant species it has been associated with under protection so far as we currently know. The webbing is viewed by the industry as "unsightly" and whether the webbing itself is facilitating any damage remains to be seen. In terms of control, Decis Protech (Bayer Crop Science UK) a deltamethrin-based insecticide has been shown to be effective at combatting older generations of *S.fractinervis* in glasshouses, but future generations appear to recolonise shortly after (pers. comm. Andrew Gaunt, PHSI/APHA). Alternatively, the Staphylinid biological control agent *Atheta* spp. which is effective against Ephydrids and Sciarids has been shown to be very effective (pers. comm. Neil Helyer, Fargro Ltd.).

## Funding

This work was supported by the UK Government's Department of Environment, Food and Rural Affairs (Defra) under the Defra-Fera long term services agreement.

## References

[B8008290] Bechev D., Koç H. (2006). Two new species of *Sciophila* Meigen (Diptera: Mycetophilidae) from Turkey, with a key to the Western Palaearctic species of the *S.lutea* Macquart group. Zootaxa.

[B8105293] Benson D. A., Cavanaugh M., Clark K., Karsch-Mizrachi I., Lipman D. J., Ostell J., Sayers E. W. (2012). GenBank. Nucleic Acids Research.

[B8033502] Bouchard J., Bouchard-Madrelle C. (2010). La forêt subalpine de Bonneval-sur-Arc (Savoie) depuis 2007: état de la population de *Sciophilabonnevalensis* n. sp. (Diptère Mycétophilide) strictement liée à *Phellinustremulae* (champignon polypore parasite du tremble), population décimée depuis 2003/4 par deux hivers rigoureux.. Bulletin de la Société Zoologique de France.

[B8268630] Chandler P (2022). Fungus gnats (Diptera: Mycetophilidae, Mycetophilinae). RES Handbooks for the Identification of British Insects.

[B8008300] Chandler P. J. (2006). The Neotropical species of *Sciophila* Meigen (Diptera, Mycetophilidae). Studia Dipterologica.

[B8033165] Chandler P. J., Pijnakker J. (2009). Tropical fungus gnats established in nurseries in the Netherlands (Diptera, Keroplatidae and Mycetophilidae. British Journal of Entomology and Natural History.

[B8033309] Chandler P. J. (2010). The South American fungus gnat *Sciophilafractinervis* Edwards, 1940 (Diptera, Mycetophilidae) present in Britain. Dipterists Digest (Second Series).

[B8039967] Edwards F. W. (1940). New Neotropical Mycetophilidae (IV) (Diptera).. Diptera). Revista de Entomologia.

[B8033183] Falk S. J., Chandler P. J. (2005). A review of the scarce and threatened flies of Great Britain. Part 2: Nematocera and Aschiza not dealt with by Falk (1991). Species Status.

[B8103882] Hebert P. D.N., Cywinska A., Ball S. L., deWaard J. R. (2003). Biological identifications through DNA barcodes. Proc. R. Soc. Lond. B.

[B8033192] Hutson A. M., Ackland D. M., Kidd L. N. (1980). Mycetophilidae (Bolitophilinae, Ditomyiinae, Diadocidiinae, Keroplatinae, Sciophilinae and Manotinae) Diptera, Nematocera. Handbooks for the Identification of British Insects.

[B8033210] Jakovlev J. (2011). Fungus gnats (Diptera: Sciaroidea) associated with dead wood and wood growing fungi: new rearing data from Finland and Russian Karelia and general analysis of known larval microhabitats in Europe. Entomologica Fennica.

[B8033249] Kurina O. (2020). New species and new records of *Sciophila* Meigen (Diptera: Mycetophilidae) from the Afrotropical Region. Zootaxa.

[B8033258] Madwar S. (1937). Biology and morphology of the immature stages of Mycetophilidae (Diptera, Nematocera. Philosophical Transactions of the Royal Society of London (B).

[B8050291] Malumphy C., Delaney M. A., Pye D., Quill J. (2010). Screening sticky traps under low magnification for adult *Bemisiatabaci* (Gennadius),*Trialeurodesvaporariorum* (Westwood) and *Aleyrodes* spp. (Hemiptera: Sternorrhyncha: Aleyrodidae). EPPO Bulletin.

[B8105284] Ratnasingham S., Hebert P. D.N. (2007). BOLD: The Barcode of Life Data System (www.barcodinglife.org). Molecular Ecology Notes.

[B8105323] Ratnasingham S., Hebert P. D.N. (2013). A DNA-based registry for all animal species: The Barcode Index Number (BIN. System. PLoS ONE.

[B8033267] Ševčík J. (2010). Czech and Slovak Diptera Associated with Fungi. Slezské zemské muzeum, (Opava).

[B8083737] Simon C, Frati F, Beckenbach A, Crespi B, Liu H, Flook P (1994). Evolution, weighting, and phylogenetic utility of mitochondrial gene sequences and a compilation of conserved polymerase chain reaction primers. Annals of the entomological Society of America.

[B8033347] Søli G, Kirk-Spriggs A. H., Sinclair B. J. (2017). Manual of Afrotropical Diptera. Volume 2. Nematocerous Diptera and lower Brachycera..

[B8033276] Zaitzev A. I. (1979). Xylophilous larvae of the subfamily Sciophilinae (Diptera, Mycetophilidae). Entomologicheskoje Obozrenije.

[B8033285] Zaitzev A. I. (1982). Fungus gnats of the genus *Sciophila* Meig. of the Holarctic. Nauka, Moscow.

[B8033295] Zaitzev A. I., Pravdin F. N. (1982). Morphoecological adaptations of insects in terrestrial associations.

